# Portable quantitative detection of serum total cholesterol using smartphone-based image colorimetry

**DOI:** 10.1016/j.clinsp.2026.101015

**Published:** 2026-05-30

**Authors:** Yufei Guo, Yonghua Zhao, Dandan Liang, Ziqi Li, Zehua Yang

**Affiliations:** a The First Hospital of Shanxi Medical University, Taiyuan, Shanxi, China; b Department of Laboratory, The First Hospital of Shanxi Medical University, Taiyuan, Shanxi, China

**Keywords:** Dyslipidemia, Total cholesterol level, RGB analysis, Smartphone detection, Quantification

## Abstract

**Objective:**

Traditional biochemical testing is limited by fixed sites, specialized equipment, and long turnaround times, severely reducing patient compliance with regular monitoring. To address the demands of routine lipid management, this study developed a simple, rapid and low-cost method for Total Cholesterol (TC) detection that enables high-throughput parallel processing of multiple samples.

**Method:**

This method synchronously identifies the RGB values of multiple pixels in an image through a mobile phone camera. All experiment procedures were strictly followed according to the guidelines of the National Committee for Clinical Laboratory Standards. Multiple performance indicators of the device were evaluated, and the results were compared for consistency with those from a fully automated clinical biochemical analyzer.

**Results:**

The TC concentration showed the strongest quantitative correlation with the green channel intensity (*G*-value). This method was preliminarily validated on a prototype smartphone. Both its detection precision and accuracy met clinical standards. The method demonstrated satisfactory linearity over 1.19–8.49 mmoL/L, and its analytical results for the limited samples in this study were highly consistent with those obtained from the automatic biochemical analyzer.

**Conclusions:**

Integrating low cost and high efficiency, this novel method has great application potential in indoor settings within primary healthcare institutions and resource-limited remote areas.

## Highlights

• The device features easy operation, high cost-effectiveness and supports simultaneous detection of multiple samples.
• Preliminary studies have verified the technical feasibility of a low-cost detection scheme combining smartphone colorimetry with a simple optical system.
• Further multi-dimensional optimization and upgrading of the detection system are required, which is expected to provide novel technical support for primary healthcare.

## Introduction

Cardiovascular Disease (CVD), as a major public health problem threatening human life and health, presents a serious situation worldwide. With the acceleration of population aging and urbanization, the absolute number of patients continues to increase.^1^^,^^2^ However, owing to the complexity and heterogeneity of CVD pathophysiological mechanisms, current treatments have significant limitations^3^ Studies have shown that abnormal serum cholesterol levels have the highest attributable risk among all modifiable risk factors for CVD^4^, ^5^, ^6^, making it one of the important targets for prevention and control. Moreover, dyslipidemia is also involved in the pathological progression of diseases such as neurodegenerative diseases and cancer.^7^ Therefore, maintaining cholesterol metabolic homeostasis is not only physiologically important, but also an important monitoring index for clinical studies and the follow-up observations of several diseases.

Total Cholesterol (TC), the sum of cholesterol in all lipoprotein fractions, is a key indicator for assessing the risk of cholesterol load.^8^ At present, the TC detection system is very complete, and it is the earliest and most effective standardization work performed in the clinical testing of lipid items. Internationally recognized reference methods mainly include the chemical method (Abell-Kendall method) and Isotope Dilution Mass Spectrometry (ID-MS method)^9^^,^^10^, and the COD-PAP enzyme-coupled reaction endpoint method is commonly used in routine clinical testing.

However, these traditional detection methods all rely on large-scale instruments and have high requirements for the professional competence of operators. Furthermore, the hospital consultation process is relatively cumbersome, which further prolongs the time required for issuing test results and ultimately leads to a significant reduction in patients' testing compliance. This issue is further confirmed by epidemiological data: over the past decade, the prevalence of dyslipidemia in China has nearly doubled, yet its early recognition rate and control rate have consistently remained at a low level.^11^^,^^12^ This phenomenon is particularly prominent in rural areas, which is obviously in direct correlation with the local shortage of medical resources.^12^, ^13^, ^14^

Against this backdrop, Point-of-Care Testing (POCT) technology, with its prominent advantages of low cost, independence from rigorous laboratory conditions and professional technical support, has effectively overcome numerous application bottlenecks plaguing traditional detection technologies.^15^^,^^16^ Commercially available POCT devices for TC assay are portable and mobile; however, they still require additional procurement of dedicated host instruments and matching specialized test strips. As disposable test strips act as recurring consumables, they substantially drive up the per-test cost.^17^ This renders such technology incapable of supporting large-scale nationwide popularization, restricting its application primarily to household or emergency scenarios.^18^ As early as 2014, researchers experimentally validated the technical feasibility of colorimetric analysis based on the RGB values of mobile terminals in the field of biochemical detection.^19^ Benefiting from an exceptionally high nationwide penetration rate as universal mobile terminals, smartphones possess inherent strengths, including user-friendly operation and powerful performance, demonstrating irreplaceable value in driving the technological innovation of colorimetric analysis and lowering the threshold of detection equipment.^20^, ^21^, ^22^

Herein, the authors have developed a facile detection method integrating smartphones with a simplified optical device, which enables the simultaneous quantitative analysis of serum TC in multiple samples using a 96-well microplate. This approach retains the inherent advantages of smartphone-based colorimetric assays, namely low cost and operational convenience, while significantly boosting the detection throughput through parallel detection with multi-well plates. Featuring exceptional cost-effectiveness and practicality, it is anticipated to serve as a novel, efficient and reliable detection tool for primary healthcare settings such as clinics and community health service stations.

## Materials and methods

### Main materials and equipment

The TC detection kit (CHOD-PAP substrate method, MeCan Biotechnology Co., Ltd.) contains three types of reagents: reagent 1, reagent 2, and a calibrator, which need to be stored at 2°–8 °C. Beckman Coulter AU5800 Automatic Biochemistry Analyzer. Human serum samples have been approved by the Ethics Committee of the First Hospital of Shanxi Medical University (license number: KYLL-2024–180)

### Introduction of smartphone-based microplate analyzer

The detection system used in this experiment is a smartphone-based microplate analyzer independently developed by the Laboratory of the School of Biological Engineering, Taiyuan University of Technology^23^ Its quantitative detection principle derives from the corollary of the Lambert-Beer law: under a stable light source, the concentration of the target analyte is inversely proportional to the reflected light intensity of the chromogenic region. The system consists of a 3D printed hardware model and a smartphone application.

**Fig. 1:**
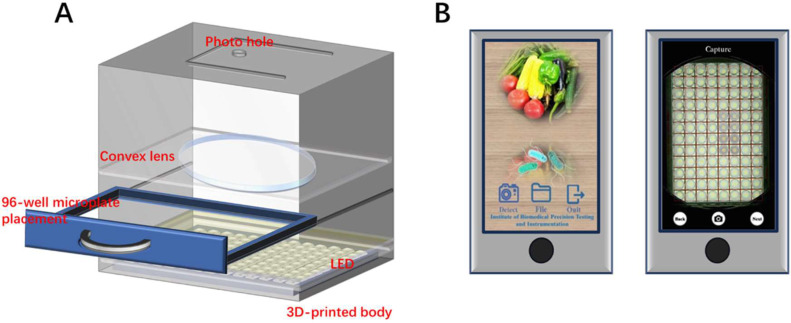
smartphone-based microplate analyzer. (A) Schematic diagram of the structure of the 3D printed hardware model; (B) Smartphone application interface: Home screen and the assay interface where microplate images are captured.

The hardware model has a fully enclosed structure, which can effectively avoid the interference of environmental light on detection results. Its core optical module is composed of a large-diameter convex lens and integrated optical devices, with a movable drawer structure adapted to 96-well microplates in the lower layer. The large-diameter convex lens ensured that the object distance from each micro-well to the smartphone camera was consistent. The accompanying light source system not only provides stable and uniform lighting conditions but also eliminates the influence of the smartphone body shadow on color information extraction via optical path optimization. The circular positioning holes designed on the top of the model not only enabled the camera to clearly capture the image of the microplate, but also prevented detection errors caused by positional deviation during shooting through mechanical limit structures. The model structure is illustrated in Fig. 1A.

The software system is developed based on the highly compatible Android platform and installed and run on a Huawei smartphone (Huawei Enjoy 7 Plus). As shown in Fig. 1B, the main operation interface is set with three major functional modules: “Detect”, “File”, and “Quit”. The detection process is as follows: After the user clicks “Detect” to enter the detection interface, they align the 12 × 8 red grid rectangle displayed on the interface with the 96-well microplate placed below. The camera of the device automatically triggers the focusing function to capture a clear image of the 25 × 25 pixel center area of the microplate. If focusing fails or there is a shooting deviation, the user can click the “Back” button to reacquire the image until satisfied, then click “Next” to proceed to the subsequent processing stage. The system automatically performs image processing and spectral analysis on the encoded pixel data, converts the color to RGB values, and draws a working curve to achieve subsequent simultaneous quantitative detection of multiple samples.

### Performance evaluation experiment

This study strictly followed the guidelines of the National Committee for Clinical Laboratory Standards (NCCLS) to conduct a comprehensive assessment of the performance of this detection system and adopted the 10% defined by CLIA'88 as the Total Error definition target (TEa).

#### Determine the reagent ratio and establish a standard curve

The ratio of sample to reagent and reaction conditions was determined through a preliminary experiment. In the formal experiment, seven concentration gradients of samples were prepared via serial dilution, and samples at each concentration level as well as blank samples, were subjected to triplicate parallel determinations. A coordinate system was constructed with the theoretical concentration values as the X-axis and the average of the measured RGB values as the Y-axis. After evaluating the experimental representativeness of each component in the RGB color model, the linear equation with the highest precision at each concentration and the coefficient of determination *R*^2^≥0.95 was selected as the standard curve for this study.

#### Assessment of detection capability

The evaluation of Limit of Blank (LoB), Limit of Detection (LoD), and Limit of Quantitation (LoQ) was conducted with an experimental protocol strictly designed in accordance with Guideline EP17-A2. During the experiment, two boxes of reagents from the same batch lot were used for testing. Finally, the larger estimated value was selected from the two sets of data as the final evaluation result. The experimental settings were both 5% for Class I error (α) and Class II error (β).

The test samples for LoB) and LoD were deionized water and four different concentration samples (with concentrations 1–4 times the LoB result), respectively. Measurements were performed continuously for 3-days (2 batches/day), yielding 60 total results. Result distribution and statistical characteristics were analyzed, and a suitable method was selected to estimate LoB and LoD.

Based on the LoD test results, the Total Error (TE) was calculated as TE=|Bias|+2*SD, where Bias is the difference between the mean of the measurements and the theoretical concentration, and SD is the standard deviation. LoQ = LoD was determined when TE was less than TEa; if TE was greater than TEa, four higher concentration samples were selected, and two batches of each concentration sample were run per day, with each batch repeated twice for 2.5 consecutive days of testing, producing a total of 40 test results. Compare the new test results with TEa. If it still does not meet the requirements, then re-select the sample concentration and repeat the above test process until TE is less than TEa, at which time the minimum concentration of the test sample is LoQ.

#### Precision evaluation

Two high- and low-test samples were prepared with concentrations close to the medically determined level. The experimental design involved shipping two batches per day, with each batch separated by more than 2 h, and each sample is subjected to a parallel double assay. The results were collected for 20 consecutive days to calculate the intra-batch, inter-batch, inter-day, and total precision of each concentration sample. The criterion for determining outliers was set at 5.5 SD obtained from the initial precision evaluation.

#### Establishment of linear range

Low-value and high-value standard samples with suitable concentration ranges were selected, mixed, and diluted according to the proportional gradient. Nine gradient concentration level samples covering high and low concentrations were prepared, and each sample was measured in parallel, twice. Taking the theoretical concentration as the X-axis and the mean value of the measured concentration as the Y-axis, linear fitting, second-order polynomial regression, and third-order polynomial regression analyses are carried out, respectively, requiring that the coefficient of determination of each fitted model (*R*^2^) should not be lower than 0.99, and the data sets should be verified by imprecision.

#### Determine the clinical reportable range

Three high-value samples close to the upper limit of the measurement interval and within the interval were selected, and gradient dilution was performed on each sample. The concentration of the diluted sample corresponding to the smallest number of dilutions should be within the measurement interval. Three replicate measurements were performed on the original sample and each of its diluted samples. The reduced concentration of each sample at different dilution ratios was calculated, and the relative deviation from the theoretical concentration was further calculated.

### Comparison of patient samples

At least 40 clinical specimens with concentrations within the linear range were collected. A fully automated biochemistry analyzer was used as the reference method (X), and a smartphone microplate analyzer was used as the test method (Y). Repeated measurements were performed for each specimen using the two-sided method, and the linear relationship of the data was intuitively observed by plotting a scatter plot. Outliers are excluded according to the rules of statistical analysis. When the correlation coefficient *r* > 0.975, it can be confirmed that the data value range covers the concentration interval of clinical concern. The formula for *r* is as follow:

$$r=\frac{\sum_{i}^{N}\left({\bar{X}}_{j}-\bar{X}\right)\left({\bar{y}}_{j}-\bar{y}\right)}{\sqrt{\sum_{i}^{N}\left({\bar{X}}_{j}-\bar{X}\right)\sqrt{\sum_{i}^{N}{\left({\bar{y}}_{j}-\bar{y}\right)}^{2}}}}$$

After meeting the requirements for statistical analysis, the least squares method was applied to fit the resulting linear regression equation. After all the above conditions were met, the predicted Bias (Bc) at the medical decision level (Xc) and its 95% confidence interval were calculated, and ½ TEa was used as the allowable error for methodological assessment. When the upper limit of the confidence interval of expected bias is less than the allowable error, or the lower limit of the confidence interval is less than the allowable error and the allowable error is less than the upper limit of the confidence interval, it is determined that the experimental method is equivalent to the reference method; otherwise, there is an unacceptable deviation between the experimental method and the reference method, and the experimental method does not meet the requirements for clinical application.

## Results & discussion

### Establish standardized experimental procedure and standard curve

Considering the concentration range of TC in the blood and the RGB value limit of reaction color development, the optimal volume ratio of sample to reagent was 1:99 after pre-experimental verification. This experiment can be completed within 10 min at room temperature. In addition, the kit uses an endpoint detection principle, which is highly compatible with the common analytical systems used in clinical laboratories, and its anti-interference capability meets the requirements for clinical applications. The assay uses a minimal amount of serum, consuming only 2.5 μL of sample in a single run.

To minimize operation-induced result errors, the operation procedures for each well of the microplate are as follows: 2.5 μL of the test sample and 165 μL of *R*^1^ reagent were added and mixed thoroughly. The mixture was incubated at room temperature for 5 min. Then, 82.5 μL of *R*^2^ reagent was added and mixed. After incubation under the same conditions for another 5 min, the color development reaction was completed. The resulting mixture was then transferred to a smartphone-based microplate analyzer for subsequent measurement and reading.

**Fig. 2:**
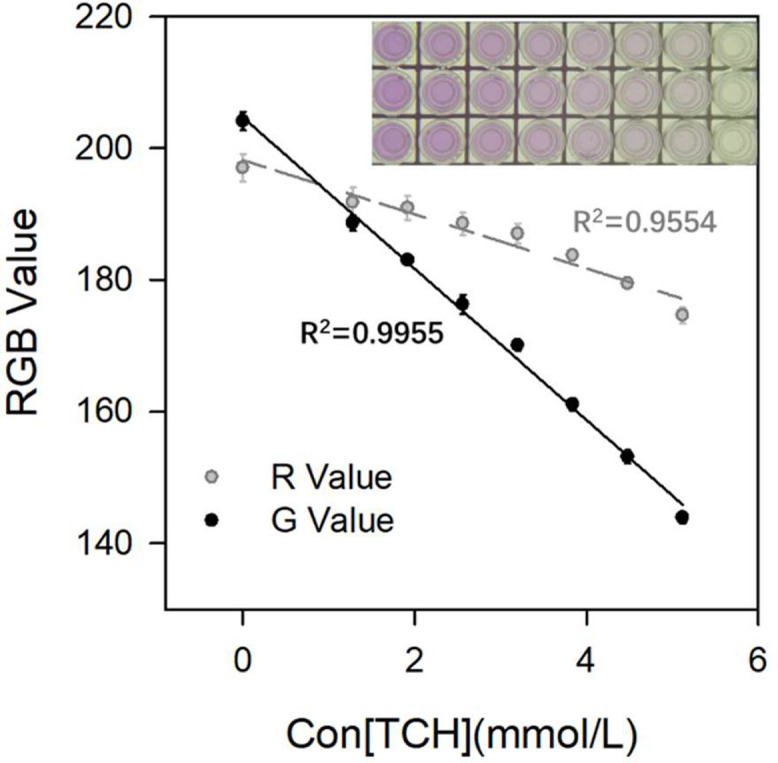
The correlation between TC concentration measured by new method and *R*-value and *G*-value respectively, and the final reaction color is located in the upper right corner of the figure.

The test results of the standard curve show that only the *R*- and *G*-values had a stable linear relationship with concentration, as shown in Fig. 2. Among them, the *G*-value had the best fit (*R*^2^ = 0.9955). Therefore, the standard curve was constructed with the *G*-value as the Y-axis, and the equation was expressed as Y = 204.7320–11.4940X.

### Assessment results of the detection capability

The test results of the blank samples were normally distributed, and the larger value of 0.36 mmoL/L was chosen as the LoB. 60-test results of LoD were non-normally distributed, and the SD of the different concentration samples fluctuated more consistently within the range of 0.04 to 0.09, so the estimation formula for LoD is: LoD = LoB + DS,β (DS,β is the value of the median of the measured values of the low concentration samples and the spacing of the 5th percentile values), and LoD = 0.93 mmoL/L was reported after calculation and comparison.

According to the above estimation of the results of the 2 batches of tests of LoD it was found that the error was greater than the TEa (Table 1). Higher level samples should be selected for LoQ estimation. 4 samples were reconstituted and tested again, and the results of both batches met the error of less than TEa. Therefore, the LoQ was reported as the minimum concentration of 1.02 mmoL/L for that batch of test.

**Table 1:** LoD and LoQ measurements of serum TC.

Project	Theoretical concentration (mmoL/L)	Average		SD		TE	
		lot 1	lot 2	lot 1	lot 2	lot 1	lot 2
LoD	1.43	1.45	1.42	0.071	0.072	16.24%	15.30%
	1.18	1.20	1.21	0.066	0.046	15.16%	12.12%
	0.92	0.91	0.88	0.095	0.085	20.08%	21.04%
	0.67	0.61	0.65	0.081	0.072	22.18%	16.30%
LoQ	1.02	0.98	1.01	0.028	0.041	9.94%	9.94%
	1.28	1.27	1.25	0.039	0.020	9.30%	6.70%
	1.54	1.54	1.54	0.021	0.039	4.30%	8.30%
	1.79	1.81	1.78	0.037	0.040	8.97%	9.66%

Batch-to-batch consistency of reagents is a critical prerequisite for ensuring the reproducibility and reliability of detection results. All inter-batch deviations in this study were within the acceptable range of experimental error. The experimental results showed that the system's LoQ was much lower than the normal serum concentration, ensuring high applicability for serum samples. In addition, colorless or pale-yellow background detection reagents were selected in the experiment. This not only avoided color signal interference but also broadened the variation range of RGB values, further enhancing the system sensitivity.

### Precision evaluation

The detection system in this study was self-developed, and its instruments and reagents were sourced from different vendors, making it inapplicable to conduct methodological evaluation using a single manufacturer’s instructions. Following the CLIA '88 quality control specifications, 1/4, 1/3 and 1/2 of TEa were set as the criteria for within-run, between-run/day-to-day and total precision in sequence. Results in Table 2 showed that at different medical decision-making levels, there were no statistically significant differences between the detection precision indicators of the two samples and their corresponding determination standards. This indicates that the precision of this system meets the clinical requirements. Notably, per the document, the tests were conducted with the same reagent batch, though results may underestimate true long-term precision. Sample stability is critical for precision evaluation when selecting test materials. Herein, processed calibrators were assayed concurrently, per the document’s preference for frozen serum samples. The precision results obtained from calibrators and serum samples were consistent, but the performance of serum samples was significantly better. This difference may be related to the instability of the calibrator after prolonged opening of the vials. Importantly, these results also demonstrate that the established method can effectively minimize matrix interference and exhibits excellent stability and reliability when analyzing real clinical patient samples. These findings also offer valuable guidance for method development, suggesting that performance assessment using real clinical samples instead of calibrators alone can better reflect the true capability of the analytical system.

**Table 2:** Precision evaluation results for calibrator and serum samples at medical decision levels.

	Sample	c (mmoL/L)	S_wr_	S_rr_	S_dd_	S_T_	DF_T_	X^2^_0.05_	X^2^_T_
Calibrator	H	5.12	1.68%	1.28%	3.32%	3.94%	28	41.3	17.2
	L	2.82	2.36%	1.42%	3.27%	4.27%	35	49.8	24.9
Serum	H	6.9	1.35%	0.37%	2.36%	2.74%	29	42.6	8.7
	L	5.21	1.98%	0.65%	2.52%	3.27%	36	51.0	15.4

### Linear range

**Fig. 3:**
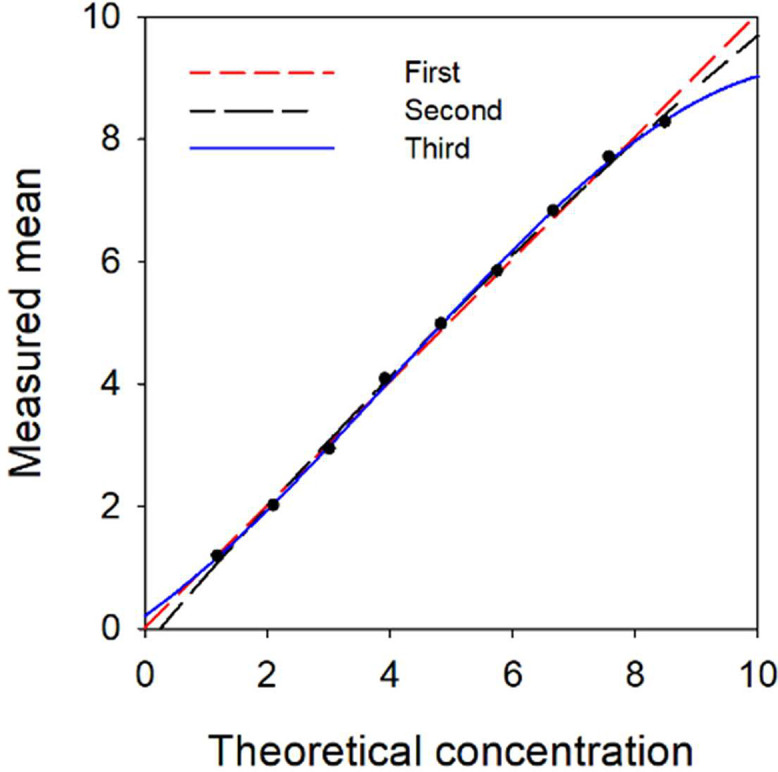
Polynomial regression analysis curves.

Serum samples with TC concentrations of 1.19 mmoL/L and 8.49 mmoL/L were mixed sequentially. The 9 mixed samples were repeatedly measured, and no outliers were found in the results (Fig. 3).

Polynomial regression analysis was performed to assess potential nonlinearity within the measurement interval (Table 3). The *t*-tests revealed that all nonlinear coefficients of the third-order model were statistically significant, and this model yielded the smallest standard error. These observations indicate that the third-order model achieved a significantly superior goodness of fit relative to other candidate models. Linear deviations at each concentration level were computed on the basis of this optimal model. The results demonstrated that the maximum deviation corresponding to all tested concentrations was substantially lower than the predefined clinically acceptable threshold. Collectively, these findings suggest that the nonlinear deviation within the measurement interval is negligible, and the detection system exhibits satisfactory clinical linearity. Meanwhile, random error analysis of the dataset revealed that repeated measurement error was relatively consistent across concentration levels and showed no proportionality to sample concentration. The imprecision was calculated at 1.93%, below the target of 1/4TEa, satisfying the testing criteria. Thus, the system’s linear range was determined to be 1.19–8.49 mmoL/L. Furthermore, the fitted curve for serum samples shows good consistency with the standard curve, enabling expansion of the detection range. This also confirmed that the assay system exhibits stable and reliable matrix effect elimination capability.

**Table 3:** Results of polynomial regression analysis.

Order	Coefficient	Degress freedom	*t*-test cut-offs	*t*-test	p	S_y.x_
First	b0	16	2.145			0.141
Second	b0	15	2.132			0.113
	b2			−2.125	>0.05	
Third	b0	14	2.12			0.076
	b2			2.436	<0.05	
	b3			−2.667	<0.05	

### Reportable range

As shown in Table 4, the three high-concentration samples showed that the diluted concentration fell within the acceptable tolerance range of the theoretical concentration at dilutions of 3-times or lower, so the maximum dilution was 3-times. The clinically reportable range was 1.02‒25.47 mmoL/L, which covers almost the entire spectrum of potential serum TC levels in patients.

**Table 4:** Dilution measurements of highly concentrated samples.

Theoretical concentration	Dilution multiple	Rep1	Rep2	Rep3	Measured mean	Reducing concentration	Deviation
8.43	2	4.28	4.01	3.90	4.06	8.13	3.57%
	3	3.28	2.99	2.89	3.05	9.15	8.60%
	4	1.86	1.89	1.78	1.84	7.37	12.60%
	5	1.70	1.56	1.62	1.63	8.13	3.56%
	6	1.29	1.09	1.23	1.21	7.23	14.20%
	7	1.15	0.96	1.14	1.08	7.56	10.28%
8.29	2	3.99	3.87	3.92	3.92	7.85	5.31%
	3	2.84	2.84	3.01	2.90	8.70	4.94%
	4	1.87	1.83	1.84	1.85	7.39	10.90%
	5	1.57	1.29	1.74	1.53	7.65	7.71%
	6	1.24	1.40	1.33	1.33	7.95	4.04%
	7	1.13	1.19	1.45	1.25	8.76	5.70%
7.68	2	3.64	3.62	3.71	3.66	7.31	4.79%
	3	2.71	2.44	2.35	2.50	7.50	2.30%
	4	1.85	2.05	2.06	1.98	7.94	3.38%
	5	1.71	1.71	2.03	1.82	9.08	18.20%
	6	1.31	1.42	1.52	1.42	8.50	10.62%
	7	1.04	1.13	1.47	1.21	8.48	10.48%

### Patient sample comparison

**Fig. 4:**
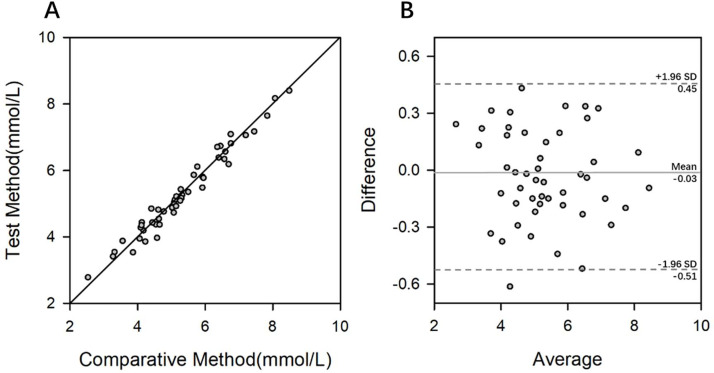
Correlation analysis results. (A) Scatter plot of the mean measurements for each method. (B) Bland-Altman plot of the means and differences between methods.

In this study, the authors followed the EP9-A2 document to perform a comparative evaluation of the two TC detection methods. The reference method was the Beckman Coulter AU5800 automatic biochemistry analyzer, and the test method was the smartphone-based microplate analyzer. A total of 48 samples were analyzed, with no outliers observed in the results. Scatter plots and bias plots (Fig. 4) demonstrated a strong linear correlation between measurements from the two systems. The calculated correlation coefficient (*r* = 0.9809) indicated that the data distribution range of the included measurement samples is suitable, enabling subsequent estimation of the slope and intercept in regression analysis. The final linear regression equation was obtained as Y = 0.95X + 0.22.

After passing the range test with a consistent scatter distribution, 4 TC concentrations were selected to estimate the prediction bias and confidence intervals. The results were presented in Table 5. At each concentration level, the expected bias was less than 1/2 TEa (5.00%), and the acceptable bias was greater than the upper limit of the confidence interval of the prediction bias. Thus, with the current sample size in this experiment, the reference method and test method exhibited comparable performance, confirming good agreement between the TC test results of the smartphone-based assay and biochemical analyzer, and the systematic error between the two methods was clinically acceptable.

**Table 5:** Expected bias of the test method at each cut-off and its acceptability.

Xc (mmoL/L)	Allowable deviations	Bc	95% CI of Bc	
			Low	High
4.0	0.20	0.02	−0.04	0.08
5.2	0.26	−0.04	−0.1	0.02
6.2	0.31	−0.09	−0.15	−0.03
7.5	0.38	−0.16	−0.22	−0.09

### Limitations

This study verified the technical feasibility of a low-cost detection strategy integrating smartphone-based colorimetry with a simplified optical system. To date, only the core methodological performance validation of the single indicator TC has been accomplished. Interference tests, matrix effect recovery tests and other relevant assays remained unimplemented, and comprehensive progress was warranted in related extended research.

#### Lipemia

With respect to the anti-interference capability of the detection system, although the reagent manufacturer provided relevant performance claims, targeted specific interference tests were not conducted for this system. Turbidity interference is a universal challenge in colorimetric detection. Distinct observations during the analysis of lipemic samples demonstrated that even after sample dilution under preset conditions, the persistent milky turbidity could lead to false deepening of the reaction solution color, thereby introducing measurement errors. Therefore, subsequent work may optimize the detection procedure by adopting a blank subtraction algorithm, so as to attenuate the interference of chylous background noise on color recognition.

#### Temperature

Temperature variation has always been a core factor in evaluating equipment performance in practical applications. The chromogenic reaction in lipid detection relies heavily on the catalysis of key enzymes. Low temperatures significantly inhibit the rate of enzymatic reactions and thus undermine the stability of detection. Considering the preset target application scenarios of the equipment, this study only verified that the detection system can achieve stable detection at a room temperature fluctuating around 17 °C. However, the equipment may still be challenged by low-temperature environments in actual use. Therefore, follow-up research is required to determine the minimum operating temperature threshold of this detection system. Support schemes shall be formulated based on test results, such as employing constant temperature accessories as optional auxiliary configurations, to improve the adaptability of this technology to diverse and complex environments.

#### Smartphone variation

This study focused on the optimization and performance validation of a detection method. Affected by the disparities in color response sensitivity of image sensors among different smartphone models^24^, the experimental validation was conducted solely with a single smartphone model, which renders the research conclusions lacking direct generalizability to other uncalibrated mobile devices. Although core imaging parameters, including exposure and white balance, were fixed during the software development stage, the hardware parameters of mobile terminals are difficult to uniformly regulate, and mobile products undergo rapid iterative updates. Further multi-model validation experiments are therefore required to clarify whether the errors induced by hardware discrepancies exceed the permissible range of the methodology. To address this limitation, this study will also consider introducing a reference-based calibration strategy to attenuate the systematic bias caused by hardware differences, thereby realizing the universal application of the detection method across diverse mobile devices.

#### Sample size

Furthermore, although the sample size of this study met the minimum requirements specified in the EP09-A2 guideline, the concentration distribution of the tested samples exhibited a clustering tendency within the medium range, while samples with extreme concentrations were sparsely distributed and lacked representativeness. This limitation weakened the validation efficacy of the detection performance in the extreme concentration range. Meanwhile, restricted by the single-center sampling design, the study fails to comprehensively and sufficiently evaluate the overall measurement error of the detection system.

### Application prospects

To continuously enhance its clinical application value and core competitiveness and align with the long-term development needs of clinical promotion, this detection system still requires multi-dimensional optimization and upgrading. On the one hand, it is imperative to overcome the limitations of existing sample cohorts. Future validation studies should targetedly augment clinical samples covering high and low extreme concentration gradients, incorporate diverse specimens encompassing distinct geographical regions and population characteristics, and conduct large-scale validation to ascertain the performance stability of the proposed method across the full concentration range. Meanwhile, multi-center collaborative studies should be conducted for further verification to clarify the procedural universality of the system under the operation of different personnel and various environmental configurations.

On the other hand, on the basis of the proven feasibility of the existing single-index detection system, the research, development and optimization of multi-index simultaneous detection technology should be prioritized. In clinical practice, the accurate interpretation of lipid metabolic status relies on the results of a complete lipid profile test. As a comprehensive lipid index, a single test result of TC is insufficient to meet the practical needs of clinical lipid screening, which is prone to missed diagnosis and misdiagnosis, thereby misleading the stratified assessment of patients’ cardiovascular disease risk and subsequent intervention decisions. Subsequent technical optimization will fully cover the indicator system for core lipid profile testing and enable the simultaneous acquisition of multi-index results with a single test, thus providing more complete and comprehensive reference data support for clinical work in primary healthcare settings.

This approach is designed to complement the advantages of a wide range of existing detection devices and establish a synergistic and efficient diagnosis and treatment system. When deployed in primary healthcare facilities as a tool for routine biochemical marker testing and clinical triage, it enables the rapid and precise identification of high-risk individuals. This, in turn, optimizes the allocation of medical resources, streamlines unnecessary referral procedures, and provides an innovative and feasible solution for the practical implementation of universal health coverage.

## Conclusion

In this work, the authors report a portable, smartphone-based colorimetric assay device for the rapid and quantitative detection of TC in human serum. The proposed method showed no clinically significant deviation from biochemical analyzer results with excellent concordance, within the scope of this preliminary feasibility study. Furthermore, its sensitivity and detection range fully meet clinical requirements. Most importantly, this novel detection method provides a rapid, simple, and cost-effective solution for complex instrumental technologies. By enabling simultaneous multi-sample testing, this assay is poised to function as an efficient and practical analytical tool for primary healthcare environments.

## Ethics approval and consent to participate

This study was approved by the Ethics Committee of the First Hospital of Shanxi Medical University, which waived the requirement for informed consent, because the serum samples used in this study were residual samples from hospital physical examination or patient examination, and informed consent could not be obtained.

## Data availability

The datasets used and/or analyzed during the current study are available from the corresponding author on reasonable request.

## Authors’ contributions

Conceptualization: Yufei Guo, Zehua Yang. Formal analysis: Yufei Guo. Funding acquisition: Zehua Yang. Investigation: Yufei Guo, Yonghua Zhao, Dandan Liang, Ziqi Li. Project administration: Zehua Yang. Resources: Zehua Yang. Supervision: Zehua Yang. Validation: Yufei Guo, Yonghua Zhao, Dandan Liang, Ziqi Li. Writing-original draft: Yufei Guo. Writing-review & editing: Zehua Yang.

## Funding

This research did not receive any specific grant from funding agencies in the public, commercial, or not-for-profit sectors.

## Declaration of competing interest

The authors declare no conflicts of interest.
